# T240. CAREGIVER BURDEN OF OUTPATIENTS WITH SCHIZOPHRENIA IN UNIVERSITY CLINIC IN SAO PAULO, BRAZIL

**DOI:** 10.1093/schbul/sby016.516

**Published:** 2018-04-01

**Authors:** Elaine Di Sarno, Isabel Cristina Napolitano, Mario Rodrigues Louzã Neto

**Affiliations:** Institute of Psychiatric, Hospital Clinics, IPQ-HC-FMUSP

## Abstract

**Background:**

The impact of schizophrenia on the family is complex1 and affects not only the patient, but his/her whole family. The adverse consequences involve physical, emotional, social, and economic restrictions and imply an objective and subjective burden for caregivers.2

This study aims to evaluate the burden of caregiving in a sample of outpatients with schizophrenia, in Sao Paulo, Brazil.

**Methods:**

Cross-sectional observational study. Patients with diagnosis of schizophrenia (DSM-5), 18–50 years, both sexes, and a relative/caregiver, both sexes, aged 18 to 70 years, living in contact with the patient at least 20 hours/week. Measures included patients and caregivers’ demographic variables. Family burden was evaluated using the Brazilian version of the Family Burden Interview Schedule (FBIS-BR), a semi-structured interview, considering objective and/or subjective burden, distributed in five subscales (assistance to the patient in daily life [objective and subjective burden]; supervision of patients’ problematic behaviors [objective and subjective burden]; financial burden; impact on family routine [objective and subjective]; worries about the patients’ present and future life [subjective]). The questions of FBIS-BR refer to the last thirty days prior to the interview, except for one item, which evaluates the overload during the last year. The objective burden is assessed in a Likert scale (1 = never to 5 = every day), and subjective burden, in Likert scale (1 = not at all to 4 = very much).

**Results:**

Patients: n= 56: 69.6% male; mean age: 36.04 ± 9.62 years; 89.3% single; duration of disease: 15.07 ± 9.83 years; number of hospitalizations: 2.95 ± 3.76; 76.8% with elementary or middle school; 66.1% without social security.

Caregivers n=56: 76.8% female; mean age: 56.30 ± 11.46 years; 57.1% mothers; 10.7 % fathers; 23.2% siblings; 57.1% married; 62.5% with elementary or middle school; in contact with the patient 81.71 ± 37.04 hours/week, most of them live with the patient; 53.6% without social security.

The mean total score of the objective and subjective burden was 2.43 ± 0.57 and 2.14 ± 0.53, respectively.

In the analysis of subscales the assistance to the patient in daily life (objective) was 3.26 ± 0.71 and it subjective aspect was 1.82 ± 0.89; supervision of patients’ problematic behaviors (objective) was 1.80 ± 0.53 and it subjective aspect was 0.95 ± 0.71. The impact on family routine (objective and subjective) was 2.21 ± 0.93 and worries about the patients’ present and future life (subjective) 3.64 ± 0.61; financial burden: 3.39 ± 1.54. The mean total family income was US$1008.49 ± $526.02.

There were no significant differences in FBIS-BR scores between male and female patients, except for “supervision of patients’ problematic behaviors”, both objective (p=.013, uncorrected for multiple comparisons) and subjective (p= .032. uncorrected for multiple comparisons) aspects, in which female patients were responsible for a higher burden for their caregivers. Regarding the family’s perception of the financial burden in the last year, 57.3% considered their spending on patients as frequent, almost always or always heavy, in the same period.

**Discussion:**

Our results are consistent with the study of Barroso et al. (2007), according to which providing care to psychiatric patients generates the feeling of overload, since the caregiver undergoes changes in his / her routine of life, failing to satisfy his / her needs to meet the needs of the patient. The burden affects almost equally male and female patients.

**References:**

1. Caqueo-Urízar A.,Castillo M., Giráldez S. et al.. Psicothema 2014: 26(2): 235–243

2. Barroso et al. Rev. Psiq. Clín 34 (6); 270–277

